# A Simple and Efficient *In Vivo* Non-viral RNA Transfection Method for Labeling the Whole Axonal Tree of Individual Adult Long-Range Projection Neurons

**DOI:** 10.3389/fnana.2016.00027

**Published:** 2016-03-18

**Authors:** César Porrero, Javier Rodríguez-Moreno, José I. Quetglas, Cristian Smerdou, Takahiro Furuta, Francisco Clascá

**Affiliations:** ^1^Department of Anatomy and Neuroscience, School of Medicine, Autónoma UniversityMadrid, Spain; ^2^Laboratorio de Vectores, Centro de Investigación Médica AplicadaPamplona, Spain; ^3^Instituto de Investigación Sanitaria de Navarra, Navarra Institute for Health ResearchPamplona, Spain; ^4^Department of Morphological Brain Science, Graduate School of Medicine, Kyoto UniversityKyoto, Japan

**Keywords:** electroporation, non-viral RNA transfection, axon tracing, Sindbis, connectomics

## Abstract

We report a highly efficient, simple, and non-infective method for labeling individual long-range projection neurons (LRPNs) in a specific location with enough sparseness and intensity to allow complete and unambiguous reconstructions of their entire axonal tree. The method is based on the “*in vivo*” transfection of a large RNA construct that drives the massive expression of green fluorescent protein. The method combines two components: injection of a small volume of a hyperosmolar NaCl solution containing the Pal-eGFP-Sindbis RNA construct (Furuta et al., 2001), followed by the application of high-frequency electric current pulses through the micropipette tip. We show that, although each component alone increases transfection efficacy, compared to simple volume injections of standard RNA solution, the highest efficacy (85.7%) is achieved by the combination of both components. In contrast with the infective viral Sindbis vector, RNA transfection occurs exclusively at the position of the injection micropipette tip. This method simplifies consistently labeling one or a few isolated neurons per brain, a strategy that allows unambiguously resolving and quantifying the brain-wide and often multi-branched monosynaptic circuits created by LRPNs.

## Introduction

Long-range projection neurons constitute a broad category defined by their axon leaving the zone where the cell soma is located to target distant regions within the brain or spinal cord. By monosynaptically linking separate local circuits into large-scale networks, LRPN cells confer the brain a functionally robust and efficient small-world network architecture (Harriger et al., 2012). Moreover, LRPN axons often give off multiple collateral branches that innervate separate brain regions. Since axon potential propagation experiences virtually no filtering at branching points (Innocenti et al., 1994; Segev and Schneidman, 1999), axons with divergent branches may bias the emergence of specific patterns of coherent activity in cell assemblies widely distributed across the brain (Jones, 2001; Dehaene et al., 2006). For these reasons, resolving LRPN axonal architectures at the single-cell level is crucial for modeling brain circuits.

Unambiguous tracing of LRPN axons requires achieving continuous labeling of a few isolated cells per brain; ideally a single one. Despite important recent advances, available methods still face important limitations. For example, intracellular filling with dyes such as biocytin or neurobiotin, that readily label the somatodendritic domain and short-range axon branches *in vivo* (Oberlaender et al., 2011) or *ex vivo* (Markram et al., 2015) fail to label axons over long distances, probably as a result of the damage inflicted to the cell soma during the intracellular procedure. Juxtacellular delivery of biocytin or dextrans (Pinault, 1996) yields a more extensive labeling of individual axons. As the juxtacellular injection involves extracellular recording through the injection micropipette, specific cell types can be targeted with precision. However, incomplete labeling is always a concern in juxtacellular injections, particularly when trying to label neurons with long and ramified axons, because of insufficient delivery of tracer, and/or possible cell damage during the injection procedure (Monconduit and Villanueva, 2005; Matsuda et al., 2009). In addition, juxtacellular injection experiments are technically demanding and low-yield. Together, these factors may explain why application of juxtacellular labeling to the study of LRPN has been relatively limited (Pinault, 1996; Prensa and Parent, 2001; Noseda et al., 2011).

Transfection with the RNA viral vector Sindbis-pal-*FP drives the rapid expression of high levels of fluorescent fusion proteins specifically directed to the axonal membrane (Furuta et al., 2001). A few years ago, it was shown that by injecting this vector into the rat brain at low titer (~10^3^ infectious units/μl) it is possible to limit the infection to a few or just one cell per brain while consistently labeling its entire axonal tree in exquisite detail (Matsuda et al., 2009). The low-titer Sindbis method has been since applied to several LPRN populations, consistently revealing an unsuspected degree of axonal extent and specificity (Kuramoto et al., 2009; Ohno et al., 2012; Aransay et al., 2015; Kuramoto et al., 2015; Nakamura et al., 2015). An important practical limitation of this method, however, is that the infecting particles drift away from the injection site for up to several 100s microns through the intercellular space before infecting a cell, thus making transfection at a given location highly unpredictable. As a result, studies of a given cell groups or nucleus requires hundreds of experiments to compile a small sample of labeled neurons of the intended type (Ohno et al., 2012; Kuramoto et al., 2015). While the encapsidated Sindbis vector has a maximal carrying capacity of about 4 kb (Nassi et al., 2015), free RNA capacity is theoretically unlimited. Besides, Sindbis viral particles require handling under P2 biosecurity conditions.

To circumvent the above limitations, we decided to examine the feasibility of achieving direct RNA transfection *in vivo* using the large, single-strand Pal-eGFP-Sindbis RNA construct while keeping it limited to one or few neurons per brain. To date, non-viral RNA transfection has been mostly carried out at the cell population level and *in vitro* (see Van Driessche et al., 2005; Yamamoto et al., 2009 for reviews). In contrast, several protocols reporting successful plasmidic DNA transfection of single cells *in vivo* have been published in recent years. However, most of such protocols require complex guidance setups such as two-photon microscopy (Kitamura et al., 2008; Marshel et al., 2010; Pagès et al., 2015), and/or patch-clamp/yuxtacellular recording (Rancz et al., 2011; Oyama et al., 2013). Recently, a simpler, “blind” protocol that combines pressure injection of plasmidic DNA and current pulses has been reported (Ohmura et al., 2015). In the present study, we attempted direct, “blind” RNA transfection of Pal-eGFP-Sindbis testing different injection methods, solution vehicles, and electric pulse sequences.

## Materials and Methods

### Animals

The brains of 81 C57BL/6 adult mice (aged 3–8 months; mean 4,8 months) of both sexes raised in our University’s animal facilities were used in this study. Procedures were carried out in accordance with European Community Council Directive 86/609/EEC and approved by our University’s Bioethics Committee. All surgical procedures were conducted under isoflurane anesthesia (0.5–2% in oxygen) following induction with a combination of ketamine (0.075 mg/g, i.p.) and xylazine (0.02 mg/g, i.p.). At the time of sacrifice, animals were overdosed with sodium pentobarbital (0.08 mg/g, i.p.).

### RNA Constructs

**FIGURE 1:**
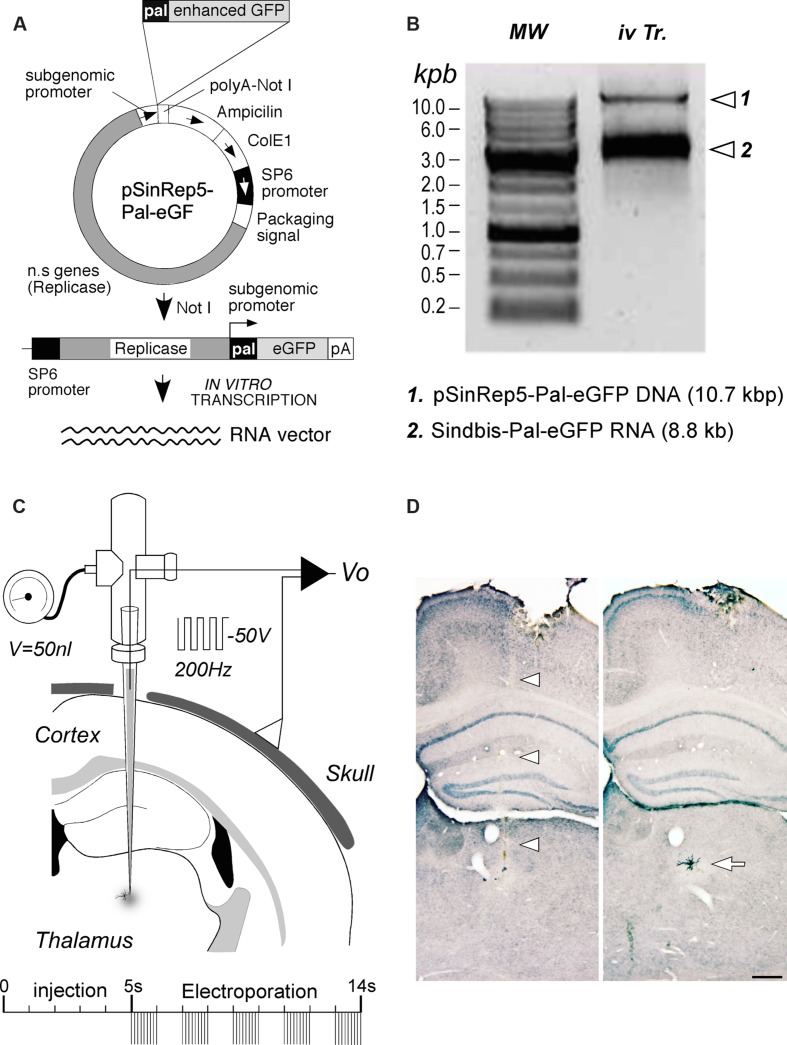
**RNA construct production and delivery. (A)** Diagram of the Sindbis-Pal-eGFP RNA production by linearization and *in vitro* transcription of the pSinRep5 template plasmid containing the pal-eGFP constructs (pSinRep5-Pal-eGFP). n.s. genes, Sindbis virus non-structural genes; pA, polyA. See text for further details. **(B)** Quality control of linearized plasmid DNA and *in vitro*- transcribed RNA on a 0.7% agarose gel. Sindbis-Pal-eGFP RNA was visualized as a single band that migrates above the 3 Kbp DNA marker. *In vitro* transcription reaction (iv Tr.) containing both the template DNA and Sindbis-Pal-eGFP RNA is shown on the right lane. DNA Molecular weight (MW) markers (Nippongenetics, Tokyo, Japan) are shown on the left lane. **(C)** Schematic diagram of the injection setup. A borosilicate micropipette back-filled by capillary action with the RNA solution is attached to a holder fitted with ports for a pressure-valve and for a wire electrode. The negative terminal of a voltage stimulator is connected to the holder electrode, and the positive terminal is attached to the skull. A 50 nl injection is followed by several trains of 1 ms and 50 V square pulses at 200 Hz. **(D)**: Brightfield images of two adjacent coronal sections showing the pipette track (arrowheads) and an isolated labeled neuron (arrow). This is the cell in **Figure 2**. GFP labeling was stabilized and intensified by nickel-enhanced immunostaining. Light thionin counterstain was applied for precise cytoarchitectonic localization of the labeling. Calibration bar: 250 μm.

An RNA construct engineered to drive the expression of an enhanced variety of the green fluorescent protein from *Aqueoria victoria* (eGFP) fusioned with a palmytoilation motif from the growth associated protein 43 (GAP43) under the Sindbis viral subgenomic promoter (Furuta et al., 2001) was used in this study. A cDNA template of the construct (pSinRep5-Pal-eGFP) was first amplified using competent *E. coli* XL1Blue bacteria. Since the functional vector is a single-strand RNA, it was necessary to make *in vitro* transcription from the plasmidic DNA (**Figure 1A**). To this end, 50 μg of plasmid were linearized by digestion with Not I restriction enzyme. After checking digestion by electrophoresis in 0.7% agarose gel, DNA templates were purified and precipitated. Finally, DNA template *in vitro* transcription was carried out using 1.5 μg of plasmidic DNA in a 50 μl distilled DEPC water solution containing 10× SP6 buffer (5 μl), rNTP mix (5 μl) (Amersham Pharmacia, UK), CAP analog (m7G(5′)ppp(5′)G) (5 μl) (New England Biolabs, Ipswich, MA, USA), Rnasin (1.5 μl) (Promega, Madison, WI, USA) and SP6 RNA polymerase (0.5 μl; New England Biolabs, Ipswich, MA, USA). After checking the synthesized Sindbis-Pal-eGFP RNA integrity on an electrophoresis gel (**Figure 1B**), this stock RNA solution (1.8–2 μg/μl) was stored at -80°C.

### Injection and Electroporation

Using a Vertical Pipette puller (Kopf, Tujunga CA, USA), micropipettes were pulled from Kwick-Fill borosilicate capillaries (1 mm of outer diameter) with an internal glass filament (WPI, Sarasota, FL, USA). Inner tip diameter was adjusted to 10–15 μm. In some experiments, narrower tips (**Table 1**) were made using a horizontal puller (Sutter Instruments, Novato, CA, USA). To eliminate RNAse activity, micropipettes were then kept in a stove overnight at 240°C. Micropipettes were backfilled with the RNA stock solution and mounted on a holder (WPI, Sarasota, FL, USA) that has both a pressure port and electrode connection (**Figure 1C**).

**Table 1:** Experimental protocols tested in the present study.

Protocols	Volume (nl)	NaCl (M)	Tip ∅ (μm)	Tip R. (MΩ)	Volt./Amp.	Frequency (Hz)	Pulse length	Total time	Cycles	N° Expts	Cases with labeling
Pr1	–	–	1–10	4–9	10–200 nA	0.5	1 s	5 s–10 m	1	9	0
Pr2	–	–	1–10	4–9	–50 V	1	50 ms	5 s	2–5	7	0
Pr3	–	–	1–10	4–9	–50 V	200	1 ms	1 s	2–5	7	0
Pr4	50–100	–	18–30	3–4	10–200 nA	0.5	1 s	5 s–10 m	1	10	0
Pr5	50–100	–	18–30	3–4	–50 V	1	50 ms	5 s	2–5	13	1
Pr6	50–100	–	18–30	3–4	–10 V	1	50 ms	5 s	2–5	5	1
Pr7	50–100	–	18–30	3–4	–50 V	200	1 ms	1 s	2–5	15	2
Pr8	50–100	–	18–30	3–4	–10 V	200	1 ms	1 s	2–5	5	0
Pr9	50–100	–	18–30	3–4	–80 V	200	1 ms	1s	2–5	5	0
Pr10	50–100	0.5	18–30	1–2	–	–	–	–	–	13	7
Pr11	50–100	0.5	18–30	1–2	–50 V	200	1 ms	1 s	2–5	28	24
Pr12	50–100	–	18–30	1–2	–	–	–	–	–	11	1

*RNA delivery protocols tested in this study. “Total time” indicates the total period for which current (on/off cycles) were applied.*

As an additional precaution to avoid contamination by RNAses, all procedures were performed over clean, single-use surfaces, surgical gloves, and masks were replaced several times during each experiment, and metal instrument tips were briefly exposed to a flame.

The anesthetized animal was placed on a Kopf stereotaxic frame (David Kopf Instruments, Tujunga, CA, USA), the sagittal midline of the scalp was sectioned and retracted, and a small craniotomy was drilled over the intended target region, usually the thalamus. The micropipette tip was positioned into the brain following the coordinates of Paxinos and Franklin (2001) stereotaxic atlas. In most protocols, 50–100 nl of solution were injected using a precision electro-valve system (Picospritzer II, Parker Hannifin, Cleveland OH). Negative current pulses were applied through the micropipette tip using a CS20 stimulator (Cibertec, Madrid, Spain). Twelve different combinations of pressure injection and/or current were tested (see Results). In most cases, an experiment was carried out in each cerebral hemisphere. After the electroporation procedure, the micropipette tip was left in place for 5 min before removing it from the brain. Finally, the bone defect was covered with a lamina of hemostatic gelfoam and the scalp was sutured. Animals were then allowed to recover from anesthesia and returned to their cages.

For control we compared our results with a number of transduction experiments (*n* = 21) with the complete Sindbis pseudoviral vector. For these experiments, the replication-defective particles were prepared by co-transfecting the same Sindbis-Pal-eGFP RNA construct (25 μl) along with a helper RNA coding for the Sindbis viral envelope and capsid proteins (25 μl), that was kindly provided to us as cDNA by Dr. Sondra Schlesinger (Columbia University, New York, NY, USA), into cultured baby hamster kidney (BHK) cells (5 × 10^6^). Particles were concentrated from the culture supernatant, titrated, and then diluted to 10^3^ particles/μl into a 0.1 M saline phosphate buffer containing 0,5% Bovine Serum Albumin. In each experiment, ~50 nl of this solution were then pressure-injected though a glass micropipette (outer tip diameter 20–40 μm).

### Histology

Because the Sindbis virus causes neuronal death beyond 72 h post-infection (Kim et al., 2004), post-injection survival in the present experiments was limited to 55–65 h. As previously reported (Furuta et al., 2001) this is time enough to achieve a complete GFP neuronal labeling. Following the survival period animals were overdosed with pentobarbital (80 mg/kg, i.p), and perfused through the left ventricle with saline (1 min), followed by 4% paraformaldehyde in 0.1 M phosphate buffer, pH 7.4, for 8 min. The brain was then removed from the skull and immersed overnight in the same fixative at 4°C. Tissue blocks were cryoprotected by soaking in a sucrose solution (30% in 0.1 M phosphate buffer) for 24 h. Serial 50-μm-thick coronal sections were obtained on a Leica freezing microtome.

**FIGURE 2:**
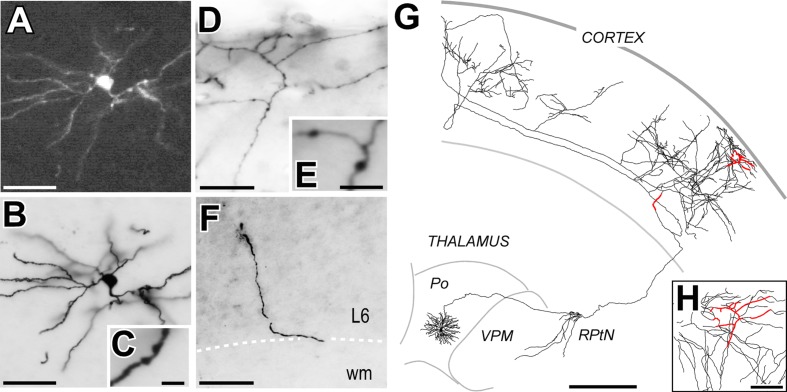
**A long-range projection (thalamocortical) cell labeled by Sindbis-Pal-eGFP free RNA transfection. (A)** Native eGFP fluorescence in the soma and proximal dendrites, as visualized in a water-mounted section. **(B,C)** The same neuron after immunostaining for GFP and ABC-DAB-Nickel intensification. **(C)** Two stubby dendritic spines are shown at high-magnification. **(D,E)** Labeled axonal branches in cortical layer 1. Axonal varicosities (putative synaptic boutons) are clearly visible at higher magnification **(E)**. **(F)** A fragment of the labeled axon at the lower border of cortical layer 6 (L6). **(G)** Camera-lucida reconstruction of the complete axonal and dendritic tree. For reference, the axonal fragments imaged in panels **(D–F)** are highlighted in red. **(H)** Higher magnification of the region outlined from **(D)**. Calibration bars: **A,B,D,F** = 50 μm; **C,E** = 5 μm; **G** = 500 μm; **H** = 100 μm.

The sections containing the area targeted in each experiment were water-mounted on glass slides and screened for the presence of GFP-expressing cells under an epifluorescence microscope (Nikon Eclipse 600) through 10–20X objectives and BV2A Nikon filter. Transfected cell somata and dendrites were brightly fluorescent (**Figures 2A,B**). However, fluorescent labeling of the axon decreased rapidly with distance, and was usually not detectable in terminal regions, probably because of the very thin caliber of the axonal tree branches and the limited sensitivity of the epifluorescence technique.

To allow intensive high-magnification microscope analysis, in those cases where transfection was successful, we made the labeling stable and opaque by using immunohistochemistry against eGFP and glucose oxidase-nickel enhacement (Shu et al., 1988) (**Figures 2B–F**). To this end, all sections were incubated, free-floating, in a rabbit anti-GFP serum (1:500; EXBIO, Prague, Czech Republic) followed by incubation with a biotinylated goat anti-rabbit serum (1:100; Sigma–Aldrich, St. Louis, MO, USA) and an avidin-biotin-peroxidase kit (1:100; Vectastain Elite, Vector Laboratories, Burlingame, CA, USA). The immunostaining not only revealed the somatodendritic morphology of the transfected cells in exquisite, Golgi like detail but also extensive distal axonal tree domains not visible with standard epifluorescence or confocal microscopy. All sections were serially mounted onto gelatin-coated glass slides and air-dried. As histological reference for the precise delineation of thalamic nuclei and cortical layers, sections were lightly counterstained with Thionin to obtain a pale Nissl-like background labeling. Finally, sections were dehydrated and coverslipped with DePeX (Serva, Heidelberg, Germany).

### Analysis of the Labeling

The immunostained sections were then systematically examined under brightfield optics at 10–40X. Since the entire axon is contained in a series of orderly mounted coronal sections, complete reconstructions can be readily carried out using camera lucida, Neurolúcida, or similar methods. The thionin counterstain allows confident delineation of nuclei and cortical layers.

To estimate the efficacy of a given transfection protocol, we calculated the percentage of cases in which any neuronal or glial cell was transfected over the total of injections carried out with that protocol. The efficacy of the various protocols was then compared using two-tailed paired Fischer’s Exact Test (*p < 0.05, **p < 0.001).

To estimate the spatial precision of the transfection, we measured the distance from the dendritic arbor of the labeled neurons to the position of the micropipette tip at the time of injection. We carried this measurement in a total of 19 cases in which was possible to recognize unambiguously the small lesion made by the tip in the same or an immediately adjacent section to that containing the labeled cell. Distances in experiments with infective Sindbis or free RNA transfection were compared (Mann-Whitney non-parametric test). All data are expressed as mean ± SD.

## Results

We devised and tested 12 different protocols aimed at achieving precisely localized transfection *in vivo* through a micropipette loaded with the Sindbis-Pal-eGFP RNA solution. Besides, as a standard control for efficacy, we also carried out a number of transduction with the complete Sindbis vector following a standard delivery protocol (Matsuda et al., 2009).

First, we attempted RNA transfection using only pulses of electric current, without pressure injection of the RNA solution into the tissue; Protocols (Pr) 1–3 (**Table 1**). Specifically, we tested the following parameter combinations: (a) low current intensity in long pulses, as routinely used for electroporating large molecules such as biotinylated dextrans into well-localized brain tissue domains (Reiner et al., 2000; Frangeul et al., 2014) (Pr1); and (b) high-voltage at two different frequencies: 200Hz (Pr2) and 1 Hz (Pr3), as in DNA plasmid electroporation protocols (Haas et al., 2001; Barnabé-Heider et al., 2008; Borrell, 2010; Ohmura et al., 2015). Despite a substantial number of trials, no labeling was observed.

Reasoning that failure by the current pulses alone to eject RNA from the micropipette could be a confounding factor for the above negative results, in the remaining protocols we systematically first ejected 50–100 nl of the RNA solution at a rate of 10 nl/s using a precision pressure injection system (Picospritzer II, Parker Hannifin, Cleveland OH). We then tested volume injections combined with delivery of different combinations of electric parameters (Pr4–Pr9, **Table 1**). In some of the experiments with the longer current pulses (Pr5 and Pr6) occasional cells were transfected (**Table 1**); however, in these experiments, an extensive electrolytic tissue lesion was evident around the position micropipette tip.

**FIGURE 3:**
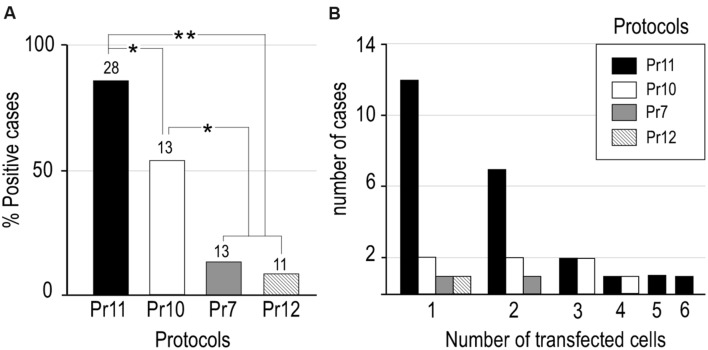
**Efficacy comparisons between different RNA transfection protocols tested in the present study. (A)** Transfection efficacy was calculated as the percentage of cases in which EGFP-expressing cells were found over the total of cases tested with that protocol (number at the base of each column). Note that combination of high NaCl vehicle and current showed the highest transfection efficiency but a high NaCl vehicle can, by itself, significantly increase transfection efficiency compared to no NaCl vehicle with or without current (two tailed paired Fischer’s Exact Test, *p < 0.05, **p < 0.001). **(B)** The number of labeled cells in each experiment varied between one and six. See **Table 1** for protocol parameters.

Based on reports that presence of salts such as NaCl in the plasmid solution can increase electroporation efficacy in mammalian cells *in vivo* (Lee et al., 2002), we decided to explore the effect of adding NaCl to our RNA solution. We mixed a 5 M NaCl solution in DEPC-treated distilled water with the base RNA solution to a final 0.5 M NaCl concentration. As might be expected, this increased ion concentration also diminished the micropipette tip resistance (**Table 1**). We then tested the effect of pressure-injecting this high-NaCl RNA solution alone (Pr10), or of doing it followed by short electric current pulses (Pr11). Remarkably, the combination of high-NaCl with electric current pulses (Pr11) produced cell transfection in a large majority of the trials (85.7%). Statistical analysis confirmed the efficacy of Pr11 to be significantly superior to the rest (*p* < 0.05 compared to Pr10; *p* < 0.01 compared to the rest of protocols; **Figure 3A**). Note that injection of high-NaCl solution alone (Pr10), seemingly caused *per se* a significant increase in transfection efficacy (53.8% of trials; *p* < 0.05 compared to Pr11 and Pr13, **Figure 3A**). In addition to the concentrations listed in **Table 1**, higher NaCl concentrations were also tested (0.8 M, *n* = 4; 1 M, *n* = 6; 1.3 M, *n* = 3); however, at these concentrations the RNA tends to precipitate and clog the pipette tip.

Finally, to explore the possible contribution of the pressure injection procedure, we tried simply injecting the base RNA solution (without added NaCl) and no electric current at all (Pr12). This procedure yielded occasional labeling in 9.1% of the trials.

In the protocols that produced a higher ratio of successful RNA transfection (Pr10, Pr11) one or two labeled cells were the most frequent outcome (**Figure 3B**). Most labeled cells were neurons (76.5% in Pr11 and 84,6% in Pr10), while the rest were glial cells. This proportion is roughly similar to that observed with the Sindbis pseudoviral vector (Furuta et al., 2001) and other DNA electroporation protocols (Ohmura et al., 2015). Successful transfection and complete axonal labeling of LRPNs with Pr11 was obtained in several thalamic nuclei and cerebral cortex (not shown).

Free RNA-transfected neurons showed somatodendritic morphologies indistinguishable from those labeled by Sindbis infection. Fluorescent eGFP labeling was present both in the somatodendritic domains and proximal axonal tree (**Figure 2**). Subsequent enhancement and stabilization of the labeling with anti-GFP immunohistochemistry revealed in Golgi-like detail the complete axonal morphology up to their terminal branches. The complete cell morphology could be subsequently reconstructed from the serial sections using a Nikon Eclipse microscope fitted with a camera lúcida system under 20–40X objectives (**Figures 2G,H**).

**FIGURE 4:**
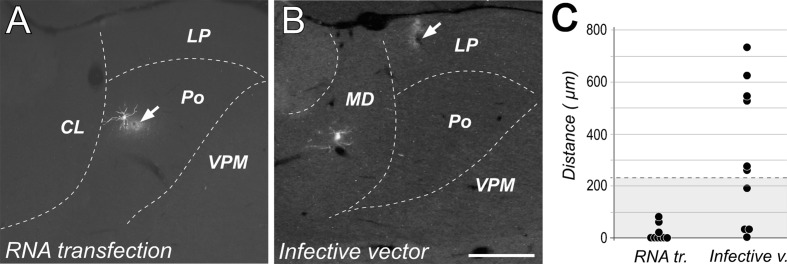
**Transfection with free RNA occurs at the tip of the injection pipette.** Native fluorescence images of cells labeled using either free RNA electroporation **(A)** or injection of infective Sindbis-Pal-GFP particles. **(B)** Note that while in **(A)** that the dendritic arbor of the labeled neuron is adjacent to the position of the micropipette tip (indicated by an arrow), the labeled neuron in **(B)** is located about 700 microns away form the injection site. Calibration bar: 500 μm. **(C)** Comparison of the distance between the tip position and the somatodrendritic domain measured for 9 neurons transfected with free RNA and 10 neurons transfected with the infective vector. For reference, the radius corresponding to the ideal sphere occupied by a volume of 50 nl (the volume of solution injected in these experiments) is shaded in gray.

In what concerns to the spatial precision of the transfection experiments, the distance between the tip position and the dendritic tree of the transfected neuron was found to be significantly lower in free RNA transfection experiments (average distance = 16.1 ± 29.6 μm) than after infective particles injections (average distance = 317 ± 266.5 μm; Mann–Whitney: *p* < 0.01; **Figure 4C**). In fact, in most of the free RNA transfection cases measured (6/9), the tip position was located within the radius of the labeled neurons dendritic tree (distance = 0 μm; **Figure 4A**), suggesting that electroporation can occur also through dendrites. This was in marked contrast with cells labeled by injections of the encapsidated vector, which were usually located at several hundreds of microns away from the position of the micropipette tip (**Figure 4B**). Only in 1 of 10 cases, the position of the tip was inside de dendritic domain of the transfected neuron. Moreover, transfection mediated by infective particles was often observed occur along the micropipette track when using the encapsidated vector probably due to solution leakage form the tip. In contrast, we never observed cells labeled along the descent of the micropipette in the free RNA experiments (**Figure 1D**).

## Discussion

We report a RNA electroporation protocol for *in vivo* transfecting single or a few adult mouse neurons that is simple, spatially precise and highly efficient. We have tested this protocol with the pal-eGFP-Sindbis RNA, which is a powerful tool for labeling and tracing the complete axonal tree of LRPNs (Matsuda et al., 2009).

We tested 12 different protocols combining different RNA delivery methods, current parameters, and the addition of NaCl to the solution on the overall efficacy of RNA transfection (**Table 1**). From these tests, we conclude that the best method is the one that combines a small volume injection of RNA suspended in a 0.5 M NaCl vehicle with the application of continuous square 1 ms current pulses at high frequency and voltage (200 Hz, 50 V). Using this protocol in a sizable number of *in vivo* adult mice experiments (*n* = 28), cell transfection (as revealed by the expression of eGFP) was achieved in 85.7% of cases. For comparison, this efficacy is as good or higher than that reported with the best published single-cell electroporation protocols of plasmidic DNA *in vivo* (Oyama et al., 2013; Ohmura et al., 2015).

The physical–chemical processes leading to the internalization of large nucleic acid molecules as a result of the exposure to electric current pulses or salt concentrations are not yet well understood (Lee et al., 2002; De Vry et al., 2010; Yuan et al., 2010; Henslee et al., 2011). However, it seems reasonable to speculate that our high transfection efficacy might result from the additive combination of several factors.

First, the high NaCl concentration diminishes about 50% the resistance of the micropipette tip and injected tissue, thus increasing the current intensity to which cells are actually exposed.

Second, our data are consistent with the interpretation that the elevated NaCl concentration might contribute to nucleic internalization by mechanisms akin to that reported for NaCl and other salts such as Calcium Chloride, Magnesium Chloride, and Calcium phosphate for plasmid-DNA transfection in cultured mammalian cells (Graham and van der Eb, 1973; Sun et al., 2013) as well as *in vivo* (Hartikka et al., 2000; Lee et al., 2002; Suzuki et al., 2003). Moreover, NaCl, Calcium chloride, and Magnesium Chloride also increase the electroporation efficacy of other polar, high-molecular weight compounds in cultured mammalian cells (Tokudome and Sugibayashi, 2003, 2004). In fact, we observed that by simply increasing the NaCl concentration in the RNA vehicle, without the application of any electric current, we increased substantially the efficacy of transfection, compared to controls (53.8% vs. 9.1%).

Third, the pressure microinjection (~50 nl) of the solution immediately before the current pulses reliably ensures the presence of an adequate concentration of RNA around the cells exposed to the current pulses.

Finally, an additional factor contributing to labeling efficacy may probably be the fact that we used a RNA construct that can self-replicate in transfected cells, leading to very high levels of a subgenomic RNA that will translate the reporter GFP gene in the cytoplasm (Furuta et al., 2001; Quetglas et al., 2010).

In recent years, several studies have described techniques for transfecting and labeling single neurons in rodent brains by DNA electroporation, but these methods require complex and expensive setups such as two-photon microscope to visualize the target cell (Kitamura et al., 2008; Marshel et al., 2010; Pagès et al., 2015) or patch-clamp/ yuxtacelular conditions to register it avoiding damage (Rancz et al., 2011; Oyama et al., 2013). Furthermore, these methods can only be applied near the surface of the brain, such as the cortex. Recently, a less demanding DNA-electroporation technique to transfect a few cells into deep brain regions has been introduced (Ohmura et al., 2015), but its efficiency is still low (50%). In contrast, our method requires only a standard pressure injection system and a stimulator setup able to deliver temporally precise trains of continuous current pulses. Moreover, the whole protocol can be implemented in a few minutes and can be readily applied to deep brain structures. An additional advantage compared to the virally mediated Sindbis transduction is that the method does not require working under P2 biosecurity conditions.

The GFP labeling obtained using the free RNA construct as described here is as rapid, intense and as complete as that obtained with the standard encapsidated Sindbis vector at very low titer (Matsuda et al., 2009) Moreover, a crucial advantage of our method is that neurons are labeled only around the micropipette tip (**Figure 4A**). In contrast, by using the encapsidated vector, cells are very frequently labeled far away from the intended injection site, making it difficult to reliably target a given brain structure (**Figure 4B**). This is a major problem in species whose brain is small in absolute terms, such as mice. We suspect that the small (60 nm) and spherical Sindbis particles (Harrison et al., 1974) drift away along the adult gray matter interstitial space for a considerable time before infecting a cell (Syková and Nicholson, 2008).

Overall, the method described here makes it simpler and more reliable the precise labeling of one or a few isolated neurons per brain *in vivo* with the highly sensitive tracer vector Pal-Sind-eGFP. Thus, it may become a valuable tool for single-cell connectomic studies of long-range projection neurons (Clascá et al., 2016).

## Author Contributions

CP, JR-M, and FC designed the study, carried out the experiments, analyzed the data, and wrote the paper. JQ and CS produced the RNA constructs and revised the paper; TF designed the vector, provided the cDNA plasmid templates and revised the paper.

## Conflict of Interest Statement

The authors declare that the research was conducted in the absence of any commercial or financial relationships that could be construed as a potential conflict of interest.

## Abbreviations

CL: central lateral thalamic nucleus
DEPC: Diethylpyrocarbonate
LP: lateral posterior thalamic nucleus
LRPN: long-range projection neurons
MD: mediodorsal thalamic nucleus
Pal-eGFP: enhanced green fluorescent protein with a palmitoylation signal
PF: parafascicular thalamic nucleus
Po: posterior thalamic nucleus
RPtN: reticular prethalamic nucleus
VPM: ventral posteromedial thalamic nucleus.
